# Calcified Thoracic Disc Herniations in the Elderly: Revisiting the Laminectomy for Single Level Disease

**DOI:** 10.1177/2192568218806274

**Published:** 2018-11-05

**Authors:** Colby Oitment, Desmond Kwok, Chris Steyn

**Affiliations:** 1 McMaster University, Hamilton, Ontario, Canada

**Keywords:** thoracic, disc herniation, myelopathy, laminectomy, thoracic compression

## Abstract

**Study Design:**

Retrospective case series.

**Objectives:**

Calcified thoracic disc herniations in the elderly present with a variety of clinical conditions and the treatment is a source of significant debate. Decompression of the disc space is done through anterior, lateral, posterolateral, and posterior approaches. There is significant morbidity of thoracic disc herniation and associated decompression.

**Methods:**

The present report is a case series of 8 elderly patients with calcified discopathy who received a simple laminectomy without decompression of the disc space.

**Results:**

Postoperatively, 5 patients mobilized independently, 2 with a walker, and 1 patient was nonambulatory. Two patients improved 1 ASIA (American Spinal Injury Association Impairment Scale) score, 1 patient improved 2 ASIA scores, and 3 patients had no change in ASIA score.

**Conclusion:**

In our experience, thoracic disc herniations require a technically difficult decompression and overall the complications are significant. We present a series of 8 patients who generally improved from a simple laminectomy and consider this a viable procedure for patients too unwell to undergo direct disc decompression.

## Introduction

Thoracic disc herniations have a bimodal distribution involving the younger patient who typically presents with acute traumatic noncalcified discopathy, and the elderly patient who has a slow degenerative clinical course and calcified disc herniation on imaging. The clinical spectrum of disease in both groups ranges from axial back pain, to radiculopathy to myelopathy. The treatment of thoracic disc disease has been a source of debate, ranging from nonoperative care (bracing, analgesics) to surgical decompression from anterior, lateral, posterolateral and posterior approaches.

Clinically, symptomatic thoracic disc herniations are rare with an incidence of 1 in 1 million patients per year^1,2^; however, more recent investigations have reported a subclinical incidence ranging between 7% and 15%.^3,4^ The earliest treatments included laminectomy for decompression of the disc space, which was associated with 59% morbidity and 13% mortality overall.^5^ Surgeons in these instances would retract the spinal cord for access to the disc space from the laminar opening posteriorly. This has led to several small published series reporting very poor outcomes. One early series reports on 3 patients, 2 of whom were paralyzed and 1 of whom died.^6^ In another series of 4 patients, 2 were paralyzed and 2 died following laminectomy for thoracic disc herniation.^7^ Other reports suggest a 75% paralysis rate and 25% death rate with aggressive retraction of the spinal cord for anterior decompression.^8^

Alternative approaches to the disc space include the transpedicular,^9^ costotransversectomy,^10^ and lateral extracavitary,^11^ all of which are associated with significant morbidity and provide limited access to central herniations.^9,10^ Transthoracic approaches and video-assisted thoracoscopy have been used with high success rates for disc decompression but are associated with higher morbidity than posterior approaches^12^ and the added complexity of the procedure. The procedure has a steep learning curve^13^ and frequent complications.^14-16^ Some researchers have suggested that central discs are better decompressed anteriorly whereas far lateral discs should be approached from posterior approaches.^17^ The specific complications encountered after thoracotomy for access to the disc space are multiple and include airway complications (increase in retained secretions, edema, increased aspiration), pulmonary complications (atelectasis, bronchospasm, pleural effusions, emphysema, chylothorax, pneumothorax), cardiac complications, hemorrhage (intercostal, segmental, or larger vessel), nerve-related complications (phrenic, intercostal, nerve root, spinal cord), and other general complications (infection, respiratory failure, requirements for chest tube with associated comorbidity).^18^

The present study examines a series of 8 myelopathic elderly patients with calcified thoracic disc herniations who received a noninstrumented laminectomy without decompression of the disc space. It is our hypothesis that patients who are medically unfit for extensive surgery or have significant medical comorbidities precluding them from direct decompression may benefit from a laminectomy without decompression of the disc space.

## Methods

This study was approved by the Hamilton Integrated Research Ethics Board (HiREB). Patients undergoing thoracic disc decompression at the Hamilton General Hospital (HGH) were reviewed between 2010 and 2017. Inclusionary criteria were (*a*) age greater than 60 years, (*b*) presence of clinical myelopathy, (*c*) presence of a calcified thoracic disc herniation, (*d*) single-level disease, and (*e*) the patient received a simple laminectomy as treatment.

Variables retrieved from medical records include demographic information, past medical history, length of symptoms prior to surgery, preoperative ASIA (American Spinal Injury Association Impairment Scale) scores and ambulation status, postoperative complications, requirements for revision surgery, postoperative clinical status (ambulation and ASIA) and mortality.

Calcification of the disc was proven either preoperatively with computed tomography (CT), X-ray, or was noted intraoperatively and mentioned on operative notes. All patients received a simple laminectomy as treatment without facetectomy or takedown of the pedicle for access to the disc space. One operative note reports subtotal discectomy via pedicle sparing approach; however, postoperative CT demonstrated no change in the disc geometry or significant decompression anteriorly so this patient was included. ASIA scores were determined by reviewing clinical notes, which listed either an ASIA score directly, or a power grading of the lower limb muscles (L2-S1). Intra- and postoperative complications were retrieved through operative notes, and separately dictated notes as well as follow-up notes.

## Results

Eight patients met inclusionary criteria and full chart review was performed (Table 1). The mean age was 74.6 years and 5 patients were female. Preoperatively, 4 patients met clinical criteria for ASIA-C, 3 for ASIA-D, and 1 for ASIA-E. Four of the patients had compression at T11-12 level, 1 at T10-11 level, 2 at T9-10 level, and 1 at T7-8 level. Associated ligamentum flavum hypertrophy was present in 5 patients. Preoperatively, 4 patients were nonambulatory, 3 mobilized with a walker, and 1 independently. The average canal compromise was 42.5%. Preoperative American Society of Anesthesiology (ASA) scores were III-IV in all patients. Onset of symptoms prior to surgery ranged from 5 days to 5 years.

**Table 1.** Demographic, Preoperative, and Postoperative Clinical Data of Included Patients.

Patient No.	Age (Years)	Sex	Preoperative ASA	Preoperative Ambulation Status	Preoperative ASIA	Level	Percentage Canal Compromise	Duration of Symptoms	Postoperative Ambulation Status	Postoperative ASIA	Mortality
1	83	Female	III	Independent	D	T7-8	50	5 years	Independent	NA	Alive
2	69	Male	IV	Walker	E	T9-10	60	1 month	Independent	E	Alive
3	82	Female	III	Nonambulatory	D	T9-10	30	2 months	Independent	E	Alive
4	72	Female	III	Nonambulatory	C	T10-11	25	5 days	Independent	E	Alive
5	63	Male	IV	Nonambulatory	C	T11-12	25	1 month	Nonambulatory	C	Deceased
6	73	Female	III	Walker	C	T11-12	50	1 month	Walker	D	Alive
7	79	Male	IV	Nonambulatory	C	T11-12	35	1 month	Independent	E	Alive
8	76	Female	III	Walker	D	T11-12	65	1 month	Walker	D	Alive

Abbreviations: ASA, American Society of Anesthesiologists score; ASIA, American Spinal Injury Association Impairment Scale score; NA, not available.

Postoperatively, 5 patients mobilized independently, 2 with a walker, and 1 patient was nonambulatory. One patient died during a complicated postoperative period from wound infection and sepsis. Postoperatively, 1 patient was ASIA-C, 2 were ASIA-D, and 4 were ASIA-E. The ASIA score was not available for 1 patient, but clinic notes indicated that ambulation was independent and the patient had improved. Two patients improved 1 ASIA score, 2 patients improved 2 ASIA scores, and 3 patients had no change in ASIA score. This information is presented in Table 1.

### Complications

One patient had an intraoperative dural tear. One patient had ongoing stenosis requiring revision decompression, which, according to operative, note was related to an intra-dural calcified disc component which was not decompressed at the original surgery. One patient did well until 2 years postoperatively when they re-presented with stenosis associated with epidural fibrosis requiring revision decompression. Two patients had a postoperative ileus and 1 patient had a urinary tract infection. Complications are listed in Table 2. Full chart review of the deceased patient revealed that the patient had a significant medical history of lymphoma with stem cell transplant, graft versus host disease, steroid-induced myelopathy, compromised immune function, and multiple other comorbidities.

**Table 2.** Complication Data Presented for Each Patient.

Patient No.	Complications
1	None
2	Ileus
3	Urinary tract infection, postoperative ileus
4	None
5	Intraoperative dural tear, postoperative surgical site infection requiring irrigation and debridement, persistent sepsis, meningitis leading to obstructive hydrocephalus and death
6	Ongoing compression, worsening of symptoms requiring revision decompression
7	Restenosis with fibrous tissue at 2 years
8	None

### Spinal Alignment

Alignment was grossly normal in all patients except 1 patient with 70° of kyphosis, and 1 patient with focal T10-L1 kyphotic deformity (patient 3). The apices ranged from T6-9 in the remainder of the patients. Alignment data is presented in Table 3 in relation to the level of compression and apex of kyphosis.

**Table 3.** Level of Compression in Relation to Degrees of Thoracic Kyphosis, Coronal Deformity, and Apex of Kyphosis.

Patient No.	Level of Compression	Degrees of Thoracic Kyphosis (T2-12)	Associated Coronal Deformity	Apex of Kyphosis
1	T7-8	19	None	T6
2	T9-10	54	None	T8
3	T9-10	55	None	T11
4	T10-11	70	None	T8
5	T11-12	49	None	T9
6	T11-12	63	None	T8
7	T11-12	8	None	T8
8	T11-12	50	Degenerative lumbar scoliosis, L2-5 30°	T8

### Location of Disc Herniation

**Figure 1.**
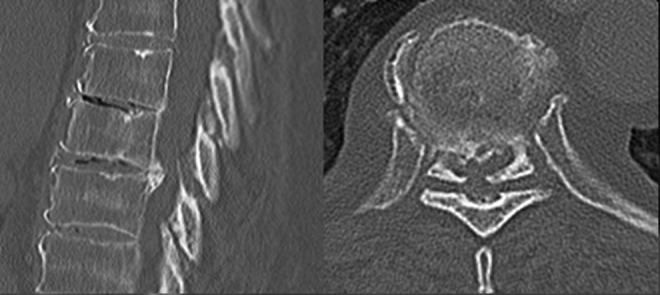
Midsagittal and associated axial computed tomographic images demonstrating a central calcified disc at T7-8 spinal level with occlusion of the canal.

**Figure 2.**
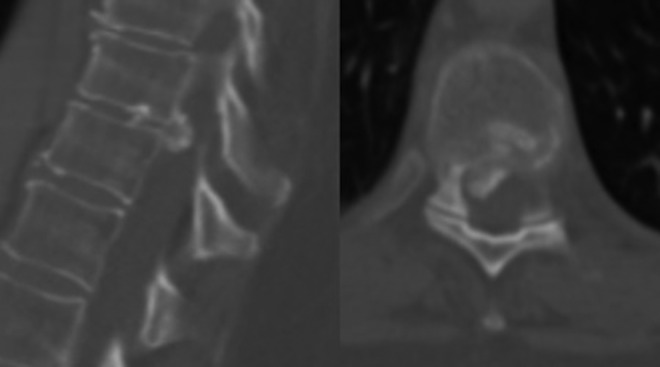
Midsagittal and associated axial computed tomographic images demonstrating a predominantly right-sided calcified disc at T9-10 spinal level with occlusion of the canal.

**Figure 3.**
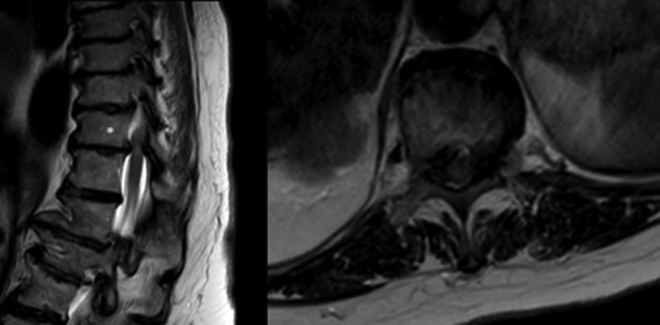
Midsagittal and axial T2 sequence magnetic resonance imaging scans of T11-12 herniated disc (T11 noted with asterisk). Compression was lateral on both sides. Calcification was demonstrated intraoperatively. Interpretation below T11 is obstructed by coronal deformity.

Three patients had predominantly lateral compression and 5 patients had central compression. Representative images of a patient with central and lateral compression are presented in CT scans in Figure 1. The classic appearance of an isolated lateral compression is represented in Figure 2. One patient had bilateral herniation, without central compression, and the magnetic resonance imaging sequences are presented in Figure 3. For this patient, CT scans were not available; however, the disc was noted to be calcified intraoperatively.

## Discussion

This study reports on a heterogeneous group of 8 elderly individuals with significant thoracic compression that received a noninstrumented laminectomy without decompression of the disc space. Some had central compression while others had unilateral compression. Results of the noninstrumented laminectomy overall are good with the majority of patients improving at least 1 ASIA scale. Aside from one patient with significant comorbidities who died postoperatively, the remainder of the patients mobilized independently or with a walker. In elderly patients with medical comorbidities such as cardiopulmonary insufficiency, the risks of direct decompression of the disc space may outweigh the potential benefit of the procedure. As far as we are aware, no studies to date examine laminectomy without disc decompression in elderly patients with calcified discopathy. It is possible, the reason for poor neurologic outcomes in early series are likely secondary cord retraction for access to the disc space rather than insufficient decompression. In our opinion, despite historically poor results, a noninstrumented laminectomy may permit satisfactory functional outcomes in high-risk patients. While minimally invasive thoracoscopic procedures are alluring, no spinal surgeons at our institution routinely perform these procedures.

In our experience, thoracic disc herniations require a technically difficult decompression and overall the complications are significant. The complication profiles in many studies may be underreported. In one series of 64 thoracic discs that were decompressed via pedicle-sparing decompression with interbody fusion only one major complication is reported, which was an epidural hematoma resulting in paralysis.^19^ Perioperative complications for costotransversectomy in other reports have complications as high as 37.5%.^11^ One series of 167 thoracoscopies reported a complication rate of 15.6%.^13^ Importantly, these complication profiles mix young healthy patients with acute traumatic discs as well as elderly patients with calcified discs. If the studies were done examining the groups separately, then rates in the elderly would be higher.

Our original hypothesis was that spinal alignment and presence of deformity would influence the outcomes of thoracic disc compression following laminectomy. Our sample size is too small to examine this relationship; however, future research should aim to determine whether greater degrees of thoracic kyphosis are associated with poorer outcomes as they increase the degree to which the spinal cord bow-strings over the disc. For this reason, apical central disc herniations would likely have worse outcomes than nonapical discs. Lateral disc herniations toward the concavity of a coronal deformity would similarly be expected to have worse outcomes than those projected toward the convexity. To date, no one has examined thoracic discs in the setting of spinal deformity.

More studies are needed to review the benefits and complication profiles of calcified thoracic discectomies in the elderly as the current complication rate is likely underestimated. Similarly, the results of laminectomy without disc decompression should be examined in the future to determine whether this is a viable option for patients who are not a candidate for direct decompression. Future studies should also aim to categorize outcomes in relation to spinal alignment and deformity as these results would likely influence decision making on an individual basis.
